# Magnetic probe-based microrheology reveals local softening and stiffening of 3D collagen matrices by fibroblasts

**DOI:** 10.1007/s10544-021-00547-2

**Published:** 2021-04-26

**Authors:** Juho Pokki, Iliana Zisi, Ester Schulman, Dhiraj Indana, Ovijit Chaudhuri

**Affiliations:** 1 Department of Mechanical Engineering, Stanford University, Stanford, CA USA; 2 Department of Electrical Engineering and Automation, Aalto University, Espoo, Finland

**Keywords:** Microrheology, Viscoelasticity, 3D cell culture, Extracellular matrix

## Abstract

Changes in extracellular matrix stiffness impact a variety of biological processes including cancer progression. However, cells also actively remodel the matrices they interact with, dynamically altering the matrix mechanics they respond to. Further, cells not only react to matrix stiffness, but also have a distinct reaction to matrix viscoelasticity. The impact of cell-driven matrix remodeling on matrix stiffness and viscoelasticity at the microscale remains unclear, as existing methods to measure mechanics are largely at the bulk scale or probe only the surface of matrices, and focus on stiffness. Yet, establishing the impact of the matrix remodeling at the microscale is crucial to obtaining an understanding of mechanotransduction in biological matrices, and biological matrices are not just elastic, but are viscoelastic. Here, we advanced magnetic probe-based microrheology to overcome its previous limitations in measuring viscoelasticity at the cell-size-scale spatial resolution within 3D cell cultures that have tissue-relevant stiffness levels up to a Young’s modulus of 0.5 kPa. Our magnetic microrheometers exert controlled magnetic forces on magnetic microprobes within reconstituted extracellular matrices and detect microprobe displacement responses to measure matrix viscoelasticity and determine the frequency-dependent shear modulus (stiffness), the loss tangent, and spatial heterogeneity. We applied these tools to investigate how microscale viscoelasticity of collagen matrices is altered by fibroblast cells as they contract collagen gels, a process studied extensively at the macroscale. Interestingly, we found that fibroblasts first soften the matrix locally over the first 32 hours of culture, and then progressively stiffen the matrix thereafter. Fibroblast activity also progressively increased the matrix loss tangent. We confirmed that the softening is caused by matrix-metalloproteinase-mediated collagen degradation, whereas stiffening is associated with local alignment and densification of collagen fibers around the fibroblasts. This work paves the way for the use of measurement systems that quantify microscale viscoelasticity within 3D cell cultures for studies of cell–matrix interactions in cancer progression and other areas.

**Supplementary Information:**

The online version contains supplementary material available at 10.1007/s10544-021-00547-2.

## Introduction

The mechanical properties of the cell microenvironment relate to a variety of diseases (Coudrillier et al. (2015); Ding et al. (2017); Holzapfel et al. 2004; Ingber (2008)). In cancer, mechanical interactions between extracellular matrix and cells affect malignancy (Dong et al. 2018; Huang and Ingber (2005); Kumar and Weaver (2009); Lee and Chaudhuri (2018)). As the matrix is generally heterogeneous at the microscale, not only structurally but also mechanically (Acerbi et al. (2015); Malandrino et al. (2018)), varying mechanical cues affecting the cells at the microscale need to be considered. Quantifying solid-tumor-relevant properties, such as the ones during developing breast cancer, necessitates for the ability to measure elasticity up to Young’s modulus (E) levels of $\mathrm{E}\geq{0.5}$ kPa. Breast tissue ranges from $\simeq$100 Pa in normal tissue to $\simeq$1–10 kPa in malignant breast-cancer tissue (Acerbi et al. (2015); Ansardamavandi et al. (2016); McKnight et al. (2002); Plodinec et al. (2012); Sinkus et al. (2007)). Current studies of how matrix mechanics impacts cells in the context of cancer progression mainly focus on static elasticity levels without considering the matrix’s time-dependent viscoelasticity, incorporating elastic and viscous, or liquid-like, characteristics (Acerbi et al. (2015); Babaei et al. (2018); Chaudhuri et al. (2014); Seewaldt (2014)). However, magnetic resonance elastography measurements have shown that breast cancer progression is associated not just with changes in stiffness or elasticity, but also with changes in loss modulus, a property related to viscous energy dissipation (Sinkus et al. (2007)). Recent studies have implicated changes in viscoelasticity as impacting various processes including stem cell differentiation, cancer cell proliferation, and cancer cell migration (Chaudhuri et al. (2016); Nam et al. (2019); Wisdom et al. (2018)). Therefore, not only sensing stiffness but also sensing viscoelasticity may play a role in impacting cancer cell phenotypes (Dong et al. (2018); Huang and Ingber (2005); Wisdom et al. (2018)). Further, sensing of matrix mechanics is a dynamic and reciprocal process, as cells can actively remodel the matrices. For example, fibroblasts contract collagen matrices, which has been reported to result in matrix stiffening at the macroscale (Bell et al. (1979); Grinnell (2000); Montesano and Orci (1988); Wozniak et al. (2003)). The dynamic cell–matrix interactions involved in cancer progression, as well as other processes, are inadequately understood.

The use of 3D cell cultures is necessary in mimicking various biological processes in vitro since cells behave differently in 3D and 2D matrices (Cukierman et al. (2001); Duval et al. (2017); Lee et al. (2019)). While 3D matrix mechanics is conventionally measured at both the macro- and nanoscales (Iwai and Uyeda (2008); Niemeyer and Adler (2002); Wisdom et al. (2018)), existing methods are unable to quantify viscoelasticity at the microscale relevant to cells within 3D cell cultures with physiologically relevant stiffness. Atomic force microscopy (AFM) and optical tweezing quantify stiffness levels of $\mathrm{E}\geq{0.5}$ kPa but measure at a surface or proximal to the surface (Blehm et al. (2016); Jorba et al. (2017); Lekka et al. (2012); Staunton et al. (2016a–b)). Particle-tracking-based passive microrheology is typically limited to materials with E<10 Pa (Jones et al. (2014)). Magnetic bead-based microrheometry can be used to probe mechanics within materials (Leung et al. (2007)), but is currently unable to measure viscoelasticity at the stiffness level of E=0.5 kPa inside 3D cell cultures using microprobes for cell-size-scale spatial resolution. Instead, larger sub-millimeter-size probes can exert forces (Furst and Squires (2017); Pokki et al. 2015; Qiu et al. (2014)) that can quantify stiffness levels of $\mathrm{E}\geq{0.5}$ kPa. Obtaining a spatial resolution comparable to cell sizes needs smaller microprobes, with a diameter of $\simeq$10 $\mu$m. However, measuring the required stiffness levels is challenging due to scaling of force with probe volume. Thus, magnetic microrheometry needs to be advanced for obtaining cell-size-scale resolution.

**Fig. 1:**
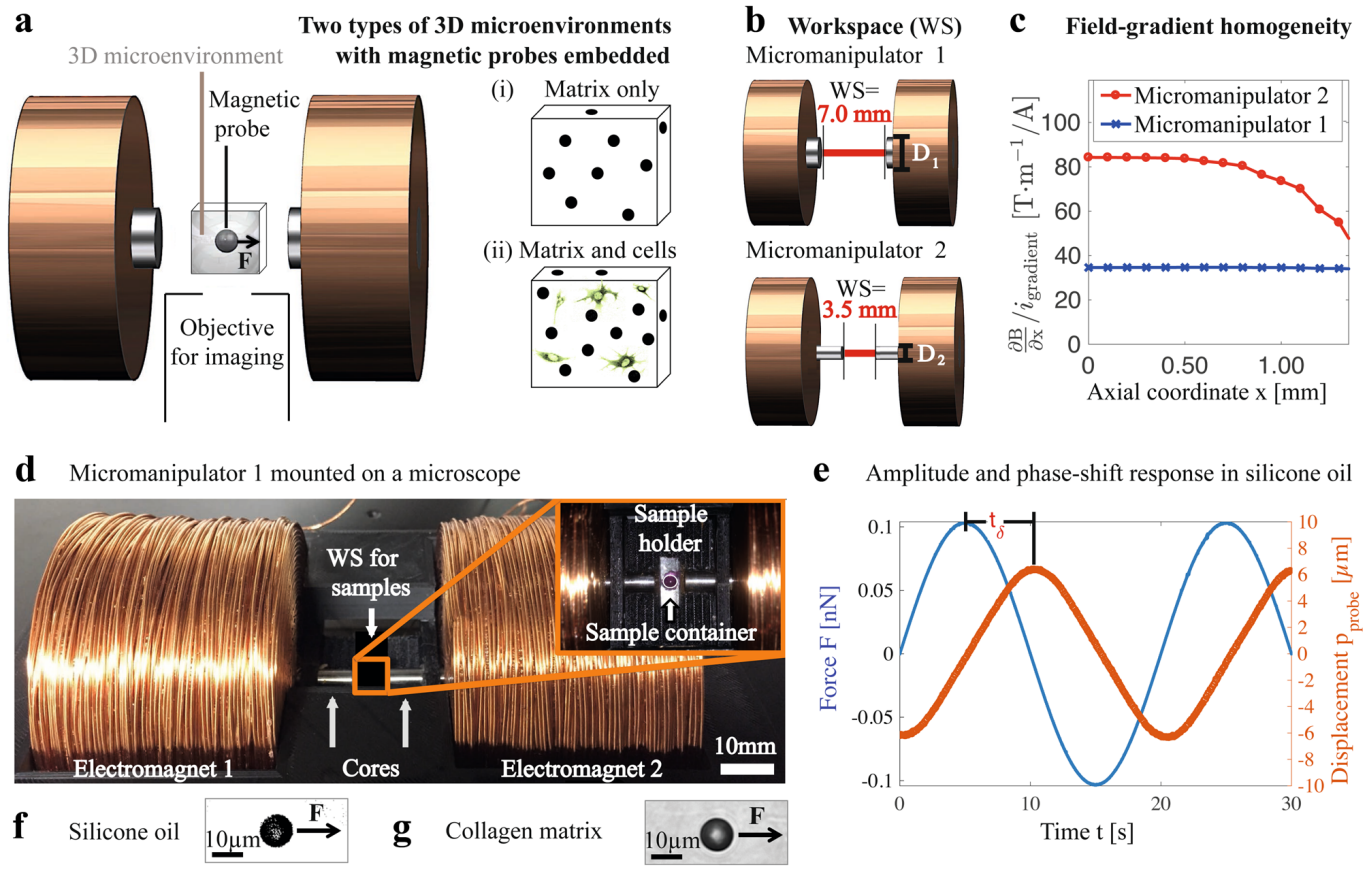
Cell-size-scale viscoelasticity in 3D microenvironments is quantified using micromanipulators and 10-$\mu$m-diameter microprobes. **a** Micromanipulator magnetically exerts forces **F** on magnetic microprobes within microenvironments of (i) matrix only, and (ii) 3D-matrix-based cell culture. Microprobes’ displacements, detected using microscopy imaging objectives, are used to extract microscale viscoelasticity. **b** Micromanipulator workspace (WS) is constrained by the separation of electromagnet cores, with diameters: D_1_=6.0 mm, or D_2_=3.0 mm. **c** Calibrated FEM simulation on magnetic-field gradient ${\frac{\partial {\textbf{B}}}{\partial x}}$ dependence on axial coordinate, normalized by *i*_gradient_. The micromanipulator 1 enables for cell-size-scale spatial resolution due to the gradient homogeneity, while the micromanipulator 2 allows for stiff-sample measurements using further increased gradients, with a decreased gradient homogeneity, thus intermediate spatial resolution. The micromanipulator 1 is the main instrument due to cell-size-scale spatial resolution. The ${\frac{\partial {\textbf{B}}}{\partial x}}$ dependence on radial coordinate is available in supplementary material. **d** Micromanipulator 1, mounted on a microscope, measures spatially varying viscoelasticity of samples with microenvironments i–ii. **e** Amplitude and phase responses to a time-dependent oscillatory force magnitude F, exerted on a microprobe, in silicone oil. The time shift t$_{\delta }$ corresponds to a phase shift of $\delta$. The oscillatory F is carried out at a frequency of f=0.05 Hz.(f–g) Silicone oil and collagen matrix were used to calibrate for the forces **F** exerted on the microprobes

Here, we report on an advancement of magnetic microrheometry for measuring the viscoelastic properties of stiffer 3D cell-culture matrices using cell-size-scale, 10-$\mu$m-diameter microprobes. These microrheometers use micromanipulators integrated to a biological microscope and consist of two tipless electromagnets to quantify 3D-matrix micromechanics (see Fig. 1a). The electromagnets and the sample workspace were designed to measure a range of Young’s moduli from 0.01 Pa to 500 Pa via sufficient magnetic-field gradients as well as via tracking of microprobes with nanometer resolution, with the goal of quantifying cell–matrix interactions. As a proof of principle, we used this tool to measure how the microscale viscoelastic properties of collagen matrices change during contraction by fibroblasts, a process that has been studied for decades at the macroscale but not the microscale (Bell et al. (1979); Grinnell (2000); Montesano and Orci (1988); Wong et al. (2012); Wozniak et al. (2003)). This contraction involves alignment of collagen-fiber bundles, and the entire strain-stiffening collagen matrix surrounding fibroblasts shrinks with time. We find that cells first soften the matrix locally, up to 32 hours of culture, and then progressively stiffen their matrix up to 72 hours of culture. Simultaneously, cells elevated the loss tangent of the matrix. The softening was found to depend on the initial cell density. The results establish the utility of the developed local magnetic-microrheometry measurements for eludicating cell–matrix interactions.

## Results

### Instrument design considerations

Magnetic microrheometers can use one or more electromagnets to apply magnetic-field gradients that exert forces on probes, and viscoelasticity is extracted based on the probes’ displacements. Although one electromagnet with a sub-millimeter tip can exert forces on cell-size-scale probes that are sufficient for measuring the stiffness levels of E=0.5 kPa (Sevim et al. (2016)), the exerted force varies non-linearly with probe–electromagnet-tip distance, and is unidirectional, hindering measurements of dynamic viscoelasticity. The use of more than one electromagnet is required for two reasons. First, two or more electromagnets configured in the appropriate geometry can generate a uniform magnetic field gradient, and thus force, over a large region (Pokki et al. (2015)), facilitating precise measurements of spatially varying viscoelasticity over that region. Second, two or more electromagnets are needed for two-directional force exertion on the probes, enabling sinusoidal oscillations of the probes as required for viscoelasticity measurements. Magnetic microrheometer systems that have these two critical properties, previously used electromagnet tips (Qiu et al. (2014)), in order to provide forces that enable for the measurements of the stiffness levels of $\mathrm{E}\geq{0.5}$ kPa. However, these tips decrease the sample workspace to the sub-millimeter level, restricting the utility of these systems for 3D cell culture gels that are typically from millimeters to centimeters in size. Here, we developed micromanipulators based on two tipless electromagnets for 3D-matrix-based cell cultures, which are capable of measuring stiffness levels up to E=0.5 kPa (see Fig. 1a). Previously, this configuration has been limited by low magnetic-field gradients (eg. 3.0 T/m at a 1 A current; Pokki et al. (2015)), and therefore, insufficient forces. Here, the micromanipulators were designed to maximize the gradients and provide sufficient forces for the measurements.

### Forces (**F**) exerted on microprobes

The micromanipulators quantify viscoelasticity within 3D microenvironments of (i) matrices consisting of pure collagen for the baseline, and (ii) collagen matrices with fibroblast cells for the actual contraction (see Fig. 1a). The micromanipulators steer microprobes in 1D using spatially controlled magnetic fields **B** [T]. These fields are generated in a cylindrical symmetric configuration: ${{ \textbf{B} }~=~\left[ {{\textbf{B}}}_{\text { x}} \ \ {\textbf{B}}_{{\text{r}}} \ \ {\textbf{B}}_{ \theta } \right] ^T}$; where *x* [mm] is the axial (on-axis) coordinate between the electromagnets, along the electromagnets’ centerline; r [mm] is the radial (off-axis) coordinate, perpendicular to the electromagnets’ centerline; and $\theta$ [rad] is the rotation angle of r around *x* axis. The electromagnets’ cobalt–iron cores concentrate the spatially controlled fields **B** on each microprobe, located at a coordinate $\mathbf{p}~=~\left[ x \ \ \text{r} \ \ \theta \right]$ (see Figs. 1b–d and supplementary material: Figs. S1–2). Due to the symmetry, the field magnitude is independent of $\theta$. A 3D-printed electromagnet/core stage and laser-cut sample holder were used for aligning all samples with the center of workspace, which is the microprobe-coordinate origin: $\mathbf{p}=\left[ 0 \ \ 0 \ \ 0 \right]$. Particularly, the forces **F** [N] applied by the spatially controlled **B** on each microprobe at the coordinate **p** are:

1 $$\begin{aligned} {\textbf{F}} = {V_{\text {mag}} \ {\nabla \textbf{B}} \ {\textbf{M} ({\textbf{B}}) }}, \end{aligned}$$

where $\mathrm{V}_{\text {mag}}$ $[\mathrm{m}^3]$ is each microprobe’s volume of magnetic material, ${{\nabla \textbf{B }}}$ is magnetic-field gradient, and **M** $[\mathrm{A} \cdot \mathrm{m}^{-1}]$ is microprobe magnetization, depending on **B**.

To extract dynamic viscoelasticity within the workspace (see Fig. 1b), sinusoidal force exertion on probes is required. Sinusoidal force generation requires positive and negative field gradients superposed on some non-zero magnetic field. Without an offset of the magnetic field, the field direction may change when switching the gradient from positive to negative, thus, a microprobe may rotate (hard magnetic) or negate its magnetization direction (soft magnetic), both, leading to distorted dynamic viscoelasticity measurements. Specifically, axial field gradients ${\frac{\partial {\textbf{B}}}{\partial x}}$, offsetted by fields $\mathrm{\textbf{B}}_{\text { x}}$, were used to exert forces **F** on microprobes. The experiments with the microprobes were carried out at the electromagnets’ center axis (see Fig. 1c), where the radial r components of ${{\nabla \textbf{B }}}$ and **B** are negligible.

### Current-based control of $\mathrm{{\nabla \textbf{B }}}$ and **B** to exert forces (**F**)

The gradients ${\frac{\partial {\textbf{B}}}{\partial x}}$ and fields $\mathrm{{\textbf{B}}}_{\text { x}}$ scale linearly for the used electromagnet currents from −2.0 to 2.0 A. Thus, ${\frac{\partial {\textbf{B}}}{\partial x}}$ and $\mathrm{{\textbf{B}}}_{\text { x}}$ were adjusted by corresponding currents, $i_{\text {gradient}}$ and $i_{\text {field}}$, respectively, as in Pokki et al. (2015). The currents $i_{\text {Electromagnet 1}}$ and $i_{\text {Electromagnet 2}}$ fed to the electromagnets were:

2 $$\begin{aligned} i_{\text {Electromagnet 1}}= & {} i_{\text {gradient}} + i_{\text {field}}, \nonumber \\ i_{\text {Electromagnet 2}}= & {} - i_{\text {gradient}} + i_{\text {field}} \end{aligned}$$

For increasing forces **F**, we optimized the micromanipulators to get a maximal level of ${\frac{\partial {\textbf{B}}}{\partial x}}$ with a sufficient spatial homogeneity, using finite-element modeling (FEM; see Figs. 1b–c). The ${\frac{\partial {\textbf{B}}}{\partial x}}$ values of the modeling were originally calibrated against experiments in Pokki et al. (2015) and here re-calibrated using hard-magnetic probe with known magnetization, density and dimensions, in viscous silicone oil, based on the force in Eq. 2 countering the force in Eq. 4 in an equilibrium. The calibrated FEM simulation enables to optimize the samples’ on-axis workspace (WS) and the optimal core diameter D$_{i}$ in which ${\frac{\partial {\textbf{B}}}{\partial x}}$ is maximized and uniform. We identified WS=7.0 mm for the micromanipulator 1, and WS=3.5 mm for the micromanipulator 2, both constrained by core-to-core separation (ie. D$_{1}$=6.0 mm, and D$_{2}$=3.0 mm, respectively; see Fig. 1b). The micromanipulator 1 has an approximately constant ${\frac{\partial {\textbf{B}}}{\partial x}}$ within the used axial dimension (see Fig. 1c), thus, a cell-size-scale spatial resolution utilizing the microprobes is acquirable. The micromanipulator 2 has a further increased ${\frac{\partial {\textbf{B}}}{\partial x}}$ magnitude that allows for the measurements of stiff samples, although an intermediate position dependency of ${\frac{\partial {\textbf{B}}}{\partial x}}$ exists (see Fig. 1c). Current-normalized ${\frac{\partial {\textbf{B}}}{\partial x}}$ magnitudes are ${\frac{\partial {{B}}}{\partial x}}$/$i_{\text {gradient}}$=34.6 $\frac{{\text {T}}}{{\text {m}}}/{\text{A}}$ for the micromanipulator 1 and ${\frac{\partial {{B}}}{\partial x}}$/$i_{\text {gradient}}$=84.6 $\frac{{\text {T}}}{{\text {m}}}/{\text{A}}$ for the micromanipulator 2 (see Fig. 1c and supplementary material: Fig. S1). Current-normalized $\mathrm{{\textbf{B}}}_{\text { x}}$ magnitudes are $\mathrm {B}_{\text { x}}$/$i_{\text {field}}$=311 mT/A for the micromanipulator 1 and $\mathrm {B}_{\text { x}}$/$i_{\text {field}}$=481 mT/A for the micromanipulator 2 (see supplementary material: Fig. S2).

### Volumetric force $\mathrm {\textbf{F}}_\mathrm {V,probe}$

Micromanipulator-based viscoelasticity measurements require knowledge of the force F exerted on each microprobe. Since the cell-size-scale, 10-$\mu$m-diameter spherical microprobes have variations of diameter D$_{\mathrm {probe}}$, we calculated volumetric force $\mathrm {\textbf{F}}_\mathrm {V,probe}=\frac{{\mathbf{F}}}{\text{V}}$, where V is each microprobe’s volume. The mean and standard deviation (SD) of microprobe diameter D$_{\mathrm {probe}}$ is: mean±SD=10.70±0.31 $\mu$m (N=51). We altered the currents, $i_{\text {field}}$ and $i_{\text {gradient}}$, to vary $\mathrm {\textbf{F}}_\mathrm {V,probe}$ applied on microprobes. The currents are constrained by the electronics’ supply of 2.0 A through each electromagnet:

$$\bullet$$   $$i_{\text {Electromagnet 1}} = i_{\text {gradient}} + i_{\text {field}} = 2.0$$ A, and

$$\bullet$$   $$i_{\text {Electromagnet 2}} = - i_{\text {gradient}} + i_{\text {field}}$$ (see Eqs. 2–b).

The maximum $\mathrm {{\textbf{F}}}_{\mathrm {V,probe}}$ magnitude ($\hat{\text {F}}_{\mathrm {V,probe}}$) maximizes the signal-to-noise ratio of the detected microprobe displacements. For the purpose, we initially distributed the microprobes in a viscous fluid, silicone oil with a dynamic viscosity of $\eta$=0.998 $\mathrm {Pa}\cdot \mathrm{s}$ at 22$^{\circ }$C. To find $\hat{\text {F}}_\mathrm {V,probe}$, we measured Stokes’ drag force ${\textbf{F}_{\text {Stokes}}}$ at low Reynolds’ numbers:

3 $$\begin{aligned} {\textbf{F}_{\text {Stokes}}}=3 \pi \mathrm{D}_{\text{probe}} \cdot \eta \cdot {\mathbf{v}}, \end{aligned}$$

where **v** is microprobe velocity. The Stokes drag force ${\textbf{F}_{\text {Stokes}}}$ counters the exerted force **F** (ie. |**F**|=|${\textbf{F}_{\text {Stokes}}}$|). The measurements yielded the $\hat{\text {F}}_\mathrm {V,probe}$ value for $i_{\text {gradient}}=1.25$ A, and $i_{\text {field}}=0.75$ A. The accuracy of $\hat{\text {F}}_\mathrm {V,probe}$ measurements is illustrated by a SD of 4.1% (see supplementary material: Fig. S3).

Next, oscillatory forces were exerted on microprobes by applying time (*t*) dependent field gradients using the current:

4 $$\begin{aligned} i_{\text {gradient}}=\hat{i}_{\text {gradient}} \cdot sin(2\pi \mathrm {f} \cdot t), \end{aligned}$$

where an amplitude of $\hat{i}_{\text {gradient}}$ = 1.25 A and a frequency of f = 0.05 Hz were set. Meanwhile, a constant level of $i_{\text {field}}~=~0.75$ A was maintained for microprobe magnetization. Particularly, the amplitude of field gradient (ie. following $\hat{i}_{\text {gradient}}$) applied on the magnetized microprobes yields $\hat{\text {F}}_\mathrm {V,probe}$. As a response to the oscillatory forces exerted on the microprobes, microprobe displacements $\mathrm {p}_{\mathrm {probe}}$ are:

5 $$\begin{aligned} \mathrm {p}_{\mathrm {probe}}=\hat{{\mathrm {p}}}_{\mathrm {probe}} \cdot sin(2\pi \mathrm {f} \cdot t - \delta ), \end{aligned}$$

where $\hat{{\mathrm {p}}}_{\mathrm {probe}}$ [nm] is displacement amplitude, and $\delta$ [ ] is the phase shift between p$_{\mathrm {probe}}$, and $i_{\text {gradient}}$ (ie. the latter is in phase with the exerted forces). The displacement amplitude $\hat{{\mathrm {p}}}_{\mathrm {probe}}$ and phase-shift $\delta$ responses in experiments using silicone oil are illustrated in Figs. 1e–f. Viscoelasticity values are reported as absolute shear modulus $|\text {G}|$ [Pa] and loss tangent ($\phi$) [ ], relying on the $\hat{{\mathrm {p}}}_{\mathrm {probe}}$ and $\delta$ responses, respectively:

6 $$\begin{aligned} |\text {G}|_{\mathrm {}}= & {} \frac{\hat{\text {F}}_{\mathrm {probe}}}{3\pi \text {D}_{\mathrm {probe}} \cdot \hat{{\mathrm {p}}}_{\mathrm {probe}}}, \nonumber \\ \phi _{\mathrm {}}= & {} \tan (\delta ) \end{aligned}$$

where $\hat{\text {F}}_{\mathrm {probe}}$ [N] is the amplitude of the exerted forces: $\hat{\text {F}}_{\mathrm {probe}}=\hat{\text {F}}_\mathrm {V,probe}\cdot \ \text{V}$.

We calibrated the micromanipulators using collagen matrices (see Fig. 1g) with varied moduli $|\text {G}|$, measured with both micromanipulators and a macrorheometer, in order to ensure the precision for stiffness measurements. This calibration provided the following $\hat{\text {F}}_\mathrm {V,probe}$ values with uncertainty information in confidence intervals (CI): the micromanipulator 1 has $\hat{\text {F}}_\mathrm {V,probe}$=1.58$\cdot$ 10$^5\frac{\mathrm {N}}{\mathrm {m}^3}$ with a CI of ±3.5 % (see supplementary material: Fig. S4a); and the micromanipulator 2 has $\hat{\text {F}}_\mathrm {V,probe}$=3.55$\cdot$ 10$^5\frac{\mathrm {N}}{\mathrm {m}^3}$ with a CI of ±4.1 % (see Fig. S4b). A modulus $|\text {G}|$ accuracy within ±4.1 % is expected for a single experiment (see supplementary material: Fig. S3).

The accuracy of the loss tangent is based on the phase shift ${\delta }$ accuracy, or time *t* accuracy with a sinusoidal force application at a frequency of f=0.05 Hz. A typical uncertainty of loss tangent, for ${\delta }$=0.38$^{\circ }$ and corresponding time value t$_{\delta }$=0.02 s, is ±0.007 (see Fig. 1e). The uncertainty for individual experiments relates to the frame acquisition speed of 20 fps with the force exertion at f=0.05 Hz, during which one frame’s acquisition corresponds a loss tangent of 0.016 (ie. ${\delta }$=0.9$^{\circ }$ or t$_{\delta }$=0.05 s).

**Fig. 2:**
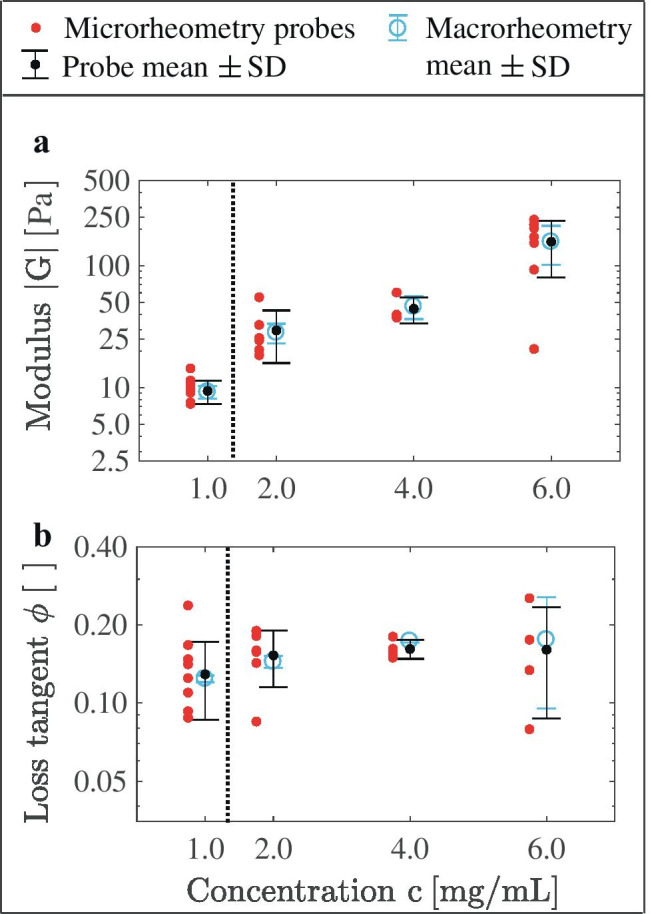
Micromanipulators can measure localized viscoelasticity. **a** Microscale modulus $\mathrm {|G|}$ and **b** loss tangent, obtained at 0.05 Hz using micromanipulator-based microrheometry, are compared with macrorheometry. The data for c=1.0 mg/mL is obtained using the micromanipulator 1 for cell-size-scale spatial resolution. The data for c=2.0–6.0 mg/mL, obtained using the micromanipulator 2, illustrates the ability to measure stiff samples

### Microscale viscoelasticity of 3D collagen matrices

Collagen matrices exhibit non-linear elasticity, or a strain-dependent elastical response, and viscoelasticity (see supplementary material Fig. S5; Chaudhuri et al. (2020); Xu et al. (2013)). Here, probe-based micromanipulators measure microscale viscoelasticity, which was altered by adjusting collagen concentration (see Fig. 2). The mean values of the viscoelasticity-describing (a) modulus $|\text {G}|$ and (b) loss tangent match between micromanipulator-based (localized) microrheometry and (bulk) macrorheometry. Wheareas the calibration (see Sec. 2.2) focuses on the mean moduli $|\text {G}|$ of varied concentrations, the matching of the mean loss tangent values validates the instrument’s accuracy for viscoelasticity measurements.

We probed localized modulus $|\text {G}|$ values up to $|\text {G}|$=239 Pa while measuring localized loss tangent. The corresponding elasticity E level relates to shear modulus G=$|\text {G}|$ with the following equation, assuming matrix isotropy:

7 $$\begin{aligned} \mathrm {E}=\mathrm {G}\cdot 2(1+\nu ), \end{aligned}$$

where $\nu$ is the matrix’s Poisson ratio. The previously measured range for this type 1 collagen is $\nu$=0.1–0.3 (Lopez-Garcia et al. (2010); Raub et al. (2010)). Thus, our maximum measured value $|\text {G}|$=239 Pa corresponds to E=525–621 Pa.

While the mean values measured using microrheometry match up with macrorheometry, variation in the measured values may reflect real spatial heterogeneity in the material. The spatial variation of viscoelasticity was further quantified for the collagen concentration of c=1.0 mg/mL. Since viscoelasticity (modulus $|\text {G}|$ and loss tangent $\phi$) varies between collagen batches, the spatial variation was analyzed with respect to each collagen batch (ie. by normalizing with the mean of the batch values: $|\mathrm{G}|_\mathrm{batch}$, and $\phi {_{\mathrm {batch}}}$). For the purpose, we quantified relative values:

8 $$\begin{aligned} |{\mathrm{G}}|^{\mathrm{rel}}_{\mathrm{probe} / \mathrm{batch}}= & {} \frac{{|\mathrm{G}|} - {|\mathrm{G}|_{\mathrm{batch}}}}{{|\mathrm{G}|_{\mathrm{batch}}}} \nonumber \\ \phi ^{\mathrm{rel}}_{\mathrm{probe} / \mathrm{batch}}= & {} \frac{\phi -\phi {_{\mathrm {batch}}}}{{\phi} {_{\mathrm {batch}}}}, \end{aligned}$$

which illustrate the spatial variation independent of the used batch (see supplementary material: Fig. S6). Particularly, the SD is 35% for $|\mathrm{G}|^{\mathrm{rel}}_{\mathrm{probe} / \mathrm{batch}}$ and 32% for ${\phi}^{\mathrm{rel}}_{\mathrm{probe} / \mathrm{batch}}$ (number of probes N=13). This quantification provides a baseline for spatial variation of viscoelasticity for the investigation of fibroblast–collagen contraction.

### Viscoelasticity during fibroblast–collagen contraction

**Fig. 3:**
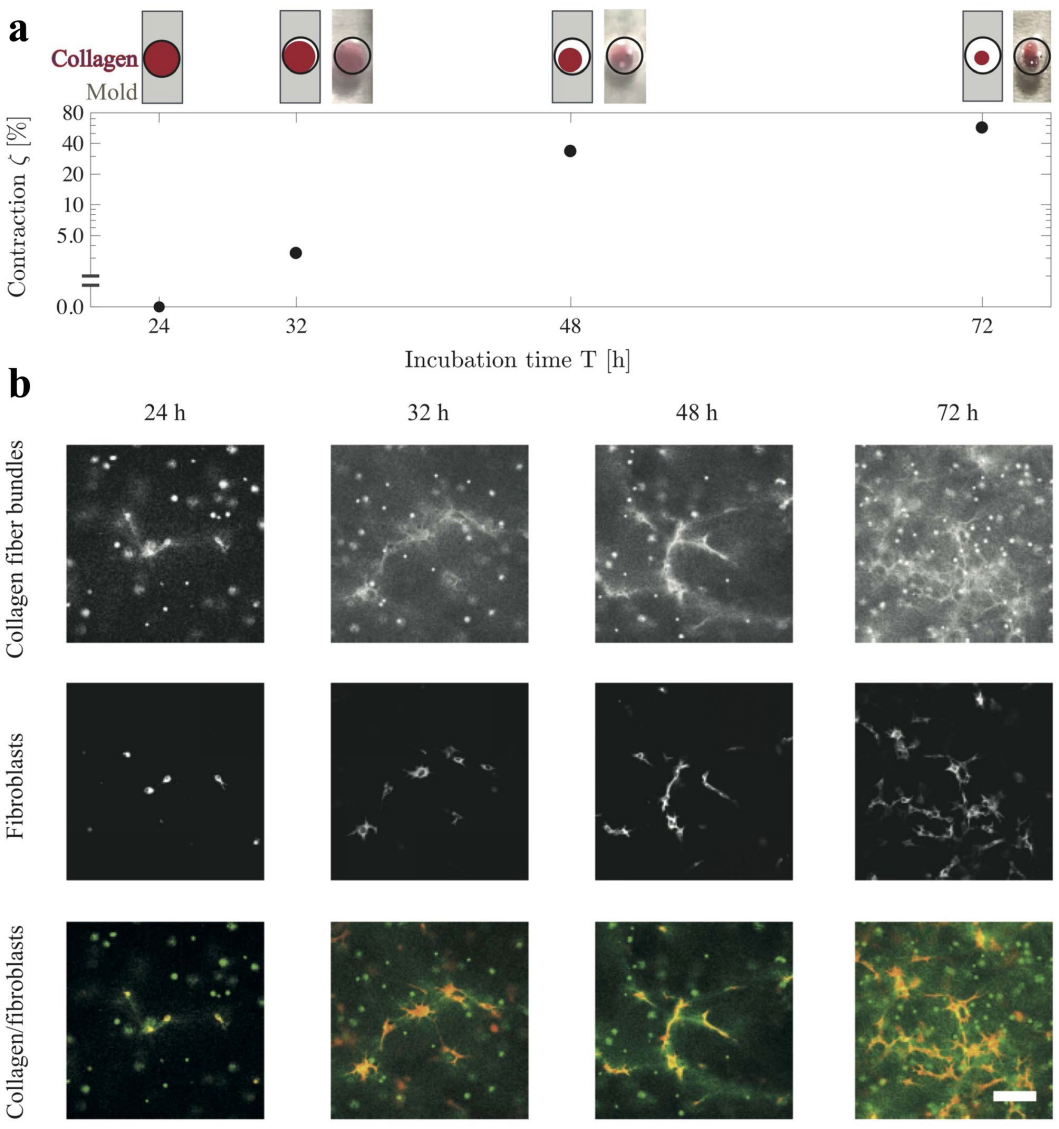
Baseline results of fibroblast–collagen contraction obtained at the level of macroscale contraction and microscale structural changes. **a** Fibroblast-induced collagen contraction, with post-measurement photos and sketches of sample cross section (diameter 3.0 mm at T=0 h). **b** Confocal images illustrate the increasing amount of collagen fiber bundles and their alignment during incubation. The top row shows confocal reflectance images of collagen fiber bundles. The middle row presents fluorescence images for the location of membrane-stained fibroblasts in respect to the collagen fiber bundles. The bottom row presents the composite images of the top and the middle rows. The scale bar denotes for 100 $\mu$m

Next, we used the magnetic probe-based microrheology to determine how fibroblasts alter the viscoelastic properties of collagen matrices during fibroblast–collagen contraction. It has previously been shown that fibroblasts continuously and extensively contract 3D collagen matrices when cultured within the matrices (Bell et al. (1979); Grinnell (2000)). Fibroblasts were cultured within 3D matrices of collagen with a concentration of c=1.0 mg/mL for 24 h, 32 h, 48 h, and 72 h. Each volume of prepared collagen was split into a matrix for experiments (with fibroblasts) and a control matrix (without fibroblasts). For an experimental baseline in relation to the literature, we quantified the macroscale contraction and the microstructural changes during incubation. The contraction, or the percentage change of sample diameter, is:

9 $$\begin{aligned} \zeta = \frac{\mathrm {D}_{0}-\mathrm{D}_{t}}{\mathrm {D}_{0}}, \end{aligned}$$

where $\mathrm{D}_{0}$ is the initial sample diameter, and $\mathrm{D}_{t}$ is the sample diameter at each incubation time. The incubated collagen samples with an initial fibroblast density of 0.5 M cells/mL experienced contraction expectedly (see Fig. 3a). The control-collagen-matrix samples had no contraction during the incubation. Complementary experiments showed that the initial cell density affects on contraction $\zeta$ at an incubation of T=32 h (ie. $\zeta <3.5 \%\text{ for 0.5 M cells/mL, and } \zeta =33\%$ for 2.0 M cells/mL). The result agrees with Montesano et al. (1988). Next, for the main experiments at the initial density of 0.5 M cells/mL, we analyzed how collagen architecture was altered over time due to fibroblast contraction using confocal microscopy (see Fig. 3b). Aligned collagen-fiber bundles were increasingly detected around the fibroblasts from 24 h to 72 h of culture time. Particularly, a densification of collagen was detected over the course of incubation. The control-matrix samples involved no detectable changes within collagen structures during the incubation. The macroscale contraction (see Fig. 3a) and the microstructural changes (see Fig. 3b) during incubation align with literature (Bell et al. (1979); Grinnell (2000)).

**Fig. 4:**
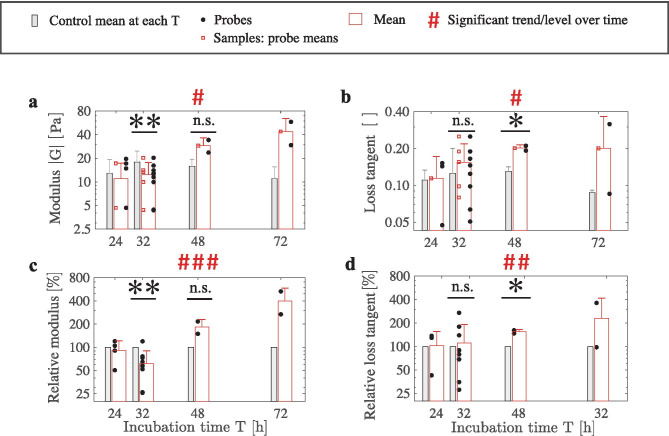
Microscale viscoelasticity measurements reveal softening and stiffening of collagen matrices during fibroblast–collagen contraction. Viscoelasticity is examined for separate incubation times (particularly the initial T=32 h), and for progressive changes between the times T. The measurements used an initial fibroblast density of 0.5 M cells/mL. **a** Measurements of the absolute shear modulus as a function of incubation time for collagen gels with fibroblasts versus control collagen gels. While an initial microscale softening of collagen matrix is found based on a reduced modulus versus control at T=32 h during minimal contraction of $$\zeta <3.5\%$$ ($$**Pr < 0.01, \text {unpaired t-test, number of cells } n=8$$), Fig. 4a progressive microscale stiffening between further incubation times is indicated by an increasing modulus ($$\#Pr~<~0.05, \text {ANOVA}, n=16$$). Analyses for absolute values are based on sample values (red squares). Red-edged bars indicate the mean values and SDs. **b** Loss tangent measurements as a function of incubation time for collagen gels with fibroblasts versus control collagen gels. While the softened matrix at T=32 h is accompanied by a loss tangent that is insignificantly increased from the control (n.s. $$Pr > 0.05, \text {unpaired t-test}, n=8$$), the loss tangent at T=48 h is significantly increased from the control ($$*Pr < 0.05, \text {unpaired t-test}, n=2$$). The level of the mean loss tangent for 24–72 h incubation is significantly elevated from the control mean level, independent of T ($$\#Pr < 0.05, \text {unpaired t-test}, n=15$$). Dependence of loss tangent on T is insignificant (n.s. Pr > 0.05, ANOVA, n=15). **c** Measurements of relative shear modulus as a function of incubation time. Probe measurements are normalized by the mean sample value. Microscale matrix softening at T=32 h is confirmed by decreased relative modulus values versus control ($$**Pr < 0.005, \text {paired t-test} , n=8$$). The matrix progressively stiffens between further incubation times ($$\#\#\#Pr < 0.001, \text {ANOVA}, n=16$$). The analysis uses probe values (bullets). **d** Measurements of relative loss tangent as a function of incubation time. While relative loss tangent is insignificantly increased in relation to control at T=32 h (n.s. $$Pr > 0.05$$, paired t-test, n=8), the loss tangent at T=48 h is significantly increased from the control ($$*Pr < 0.05, \text {paired t-test}, n=2$$). Relative loss tangent increases progressively during incubation ($$\#\#Pr < 0.005, \text {ANOVA}, n=15$$; analysis is based on mean values)

**Fig. 5:**
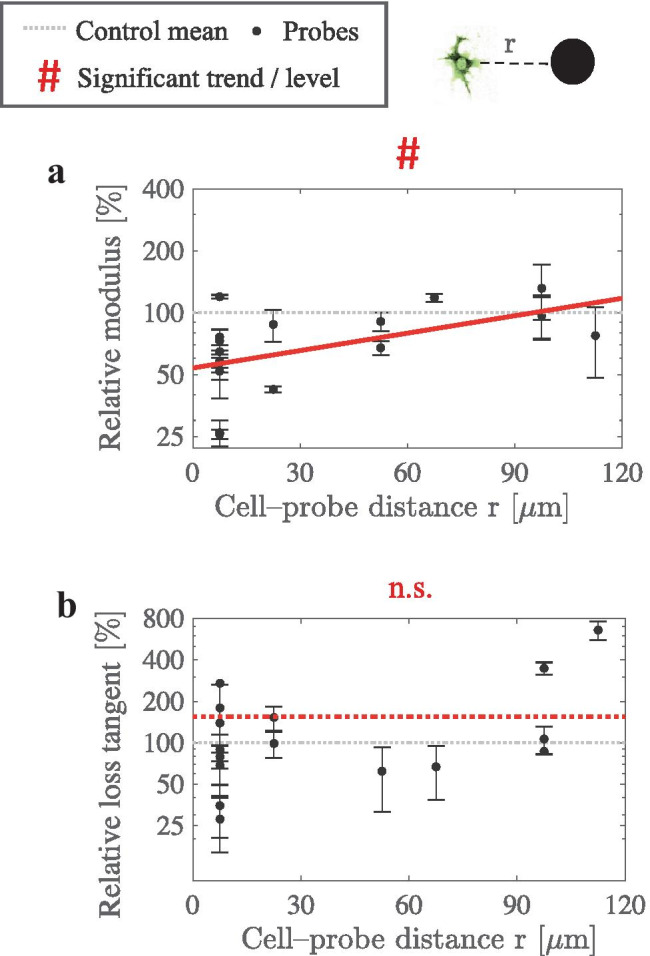
Softening is dependent on cell–probe distance at T=32 h. Spatially varying viscoelasticity was measured for the softened matrix of fibroblasts with an initial density of 0.5 M cells/mL. **a** Relative modulus is significantly below the control value at the cell–probe distance of r < 15 $\mu$m, and increases significantly with r ($*Pr < 0.05$, Pearson’s test, n=17; trend shown as solid red line). Thus, a softening gradient inward to the fibroblast cells exists. **b** The mean loss tangent has spatially an insignificantly elevated level (n.s. Pr > 0.05, unpaired t-test; n=16; dotted red line), and an insignificant trend against r (n.s. Pr > 0.05, Pearson’s test, n=16). SDs are indicated by the error bars

### Microscale viscoelasticity altered by incubation time

We measured the microscale viscoelasticity of the collagen matrix within a maximum radius of 15 $\mu$m from fibroblasts at the incubation times of 24 h, 32 h, 48 h, and 72 h. The results for the main experiments at an initial fibroblast density of 0.5 M cells/mL are shown in Fig. 4a–d. Both, the absolute (see Fig. 4a–b) and relative (see Fig. 4c–d) viscoelasticity values were compared to the ones of control samples without fibroblasts (ie. modulus $|\mathrm{G}|_{\mathrm{control}}$ and loss tangent $\phi _{\mathrm {control}}$ are mean values of each control sample). The mean±SD values of $|\mathrm {G}|_{\mathrm{control}}$ and $\phi _{\mathrm {control}}$ were 16.05±6.72 Pa (N=25) and 0.133±0.082 (N=24), respectively.

Moduli $\mathrm {|G|}$ and loss tangents, exhibited alterations during the incubation (see Figs. 4a–b). We focus on the initial matrix softening that is unexpected, due to the contraction. The softening is based on the decreased modulus $\mathrm {|G|}$ at T=32 h versus control $|\mathrm {G}|_{\mathrm{control}}$ (see Fig. 4a), and this result depends on the fibroblasts’ initial density (see details in Subsec. 2.8). During further incubation, the modulus $\mathrm {|G|}$ increases (matrix stiffens) that is expected due to the macroscale contraction, and therefore densification, of the strain-stiffening collagen (Motte and Kaufman (2013); see Fig. 3a). Besides, the mean loss tangent values ($\phi$) during the incubation are significantly elevated from the control values (${\phi}_{\mathrm{control}}$; see Fig. 4b).

To account for variations in viscoelasticity between separate collagen gel preparations, we examined relative viscoelasticity. The calculation preserves all microprobe-based measurements for sample-independent comparisons, increasing statistical power. Therefore, each modulus and loss-tangent measurement was normalized by the control mean values to obtain relative modulus and relative loss tangent, respectively:

10 $$\begin{aligned} |\mathrm{G}|^{\mathrm{rel}}_{\mathrm{probe} / \mathrm{control}}= & {} \frac{|\mathrm{G}|}{|\mathrm{G}|_{\mathrm{control}}} \nonumber \\ \phi ^{\mathrm{rel}}_{\mathrm{probe} / \mathrm{control}}= & {} \frac{\phi }{\phi {_{\mathrm{control}}}} \end{aligned}$$

Recalculating the previously-described statistical analyses (see Figs. 4a–b) using this relative viscoelasticity (see Figs. 4c–d) confirmed the results on matrix softening/stiffening and on the accompanying increased loss tangent. Further statistical analysis is based on these relative viscoelasticity values.

### Initial microscale softening of collagen by fibroblasts at an initial density of 0.5 M cells/mL

Initially, fibroblasts significantly soften the collagen matrices at the incubation time of T=32 h, prior to stiffening the matrices at the further incubation times (see Figs. 4a, c). During this softening at T=32 h, matrix contraction is minimal (ie. $\zeta <3.5\%$), and a contraction of $\zeta$=33–57% is only observed at the further incubation times (see Fig. 3a). The matrix softening was accompanied by an elevated loss tangent at T=24 h–72 h (see Fig. 4b). Particularly, a gradually increasing relative loss tangent during incubation was indicated (see Fig. 4d).

Next, we quantified the spatial gradient of viscoelasticity at the incubation time of T=32 h (see Figs. 5a–b) to comprehensively investigate the initial softening of the collagen matrix by fibroblasts. We analyzed the dependency of relative modulus and relative loss tangent, on cell–probe distance r. A significant dependency between relative modulus and the distance r indicates a softening gradient inward to the fibroblast cells. An elevated level of the mean relative loss-tangent values at T=32 h was statistically insignificant at the 95% confidence level. The presence of the micromechanical gradient around the fibroblasts suggests that local activity of the fibroblasts is leading to the softening of the matrix.

**Fig. 6:**
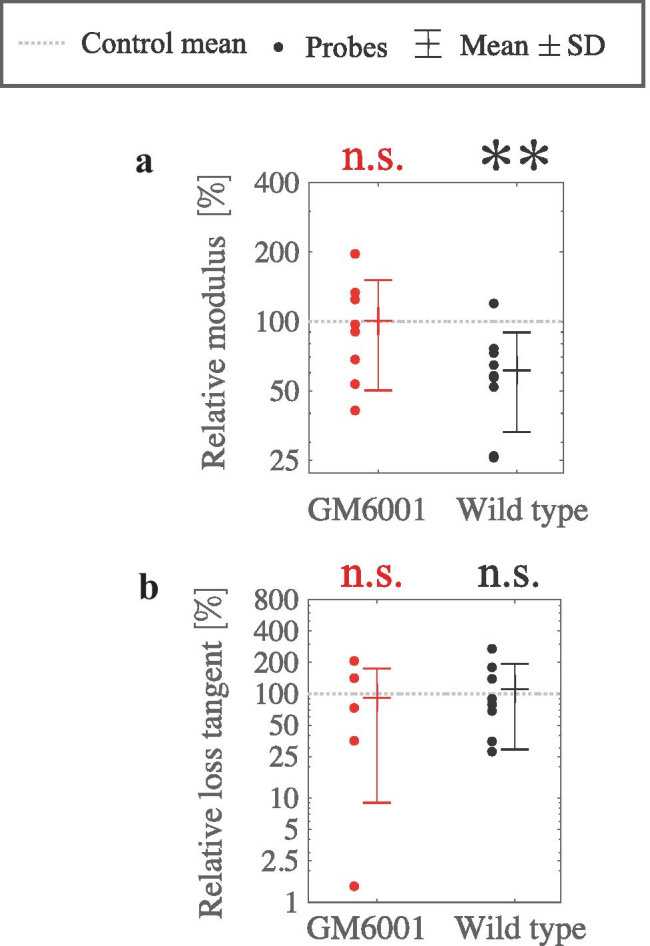
Inhibiting cellular MMP production eliminated the softening of collagen matrices of fibroblasts with an initial density of 0.5 M cells/mL at T=32 h. MMP-production of fibroblasts was inhibited using GM6001 treatment. **a** Whereas relative modulus of matrices with wild type cells is significantly lower than the one of the control matrices with collagen only (ie. softer; $$**Pr < 0.01$$, paired t-test, Bonferroni correction, n=8), the relative modulus of matrices with GM6001-treated cells has an insignificant difference from the one of the control (ie. equally stiff; n.s. Pr > 0.50, paired t-test, Bonferroni correction, n=8). **b** Relative loss tangent of matrices with GM6001-treated and wild type cells insignificantly varies from the ones of control matrices (n.s. $$Pr > 0.50$$, paired t-test, Bonferroni correction, n=5 for the treated, n=8 for wild type)

It is known that fibroblasts can secrete proteases, including matrix metalloproteinases (MMPs), to degrade the matrix, indicating protease activity to be one potential source for matrix softening. Thus, we examined the role of proteases in mediating softening of the collagen matrix at the incubation of T=32 h. We used a broad-spectrum protease inhibitor GM6001 with a tested protocol to inhibit protease activity and assess the impact on local matrix softening (Sabeh et al. (2009); Schoumacher et al. (2010); Wisdom et al. (2018)). The relative modulus and relative loss-tangent values between collagen matrices of GM6001-treated and wild type fibroblasts were statistically analyzed (see Fig. 6). The significant softening of collagen matrices of wild type fibroblasts disappeared in the matrices of GM6001-treated fibroblasts (see Fig. 6a). Similarly, we compare the relative loss-tangent values of the matrices with GM6001-treated and wild type fibroblasts (see Fig. 6b). The value of the relative loss tangent in the matrices with GM6001-treated fibroblasts is not significantly different from the control. Lastly, we compared these results at an initial density of 0.5 M cells/mL to complementary results at an increased initial density of 2.0 M cells/mL (see supplementary material: Fig. S6). The collagen-matrix softening at T=32 h disappears with the increased initial cell density.

## Discussion and conclusions

We developed magnetic probe-based microrheometers, each using a magnetic micromanipulator integrated to a microscope, which measure matrix viscoelasticity within 3D cell cultures, having Young’s modulus levels up to E=525 Pa. Therefore, these microrheometers quantify solid-tumor-relevant 3D-cell-culture viscoelasticity at the cell-size-scale spatial resolution, which has been unexplored previously. These microrheometers can map spatially varying viscoelasticity inside the 3D cell cultures unlike optical tweezers and AFM, which measure mechanics only proximal to the material surface (Lekka et al. (2012); Staunton et al. (2016a); Weihs et al. (2006)). However, the microrheometers still need further development in measuring stiff tumors with levels of E>10 kPa that can already be measured using AFMs (Bouchonville et al. (2016)). To show the applicability of our magnetic microrheometers to 3D-cell-culture measurements, we quantified how the microscale viscoelasticity of collagen matrices is altered during collagen–fibroblast contraction, a process that has been extensively studied at the macroscale. Interestingly, we found that fibroblasts first softened the matrix locally over the first 32 h, and then stiffened it, when seeded in a density of 0.5 M cells/mL. This finding was accompanied by probe–cell distance dependent softening until 32 h of incubation. However, the softening disappeared with an increased initial cell density. These new observations, not reported in previous studies of fibroblast contraction of collagen, highlights the utility of this instrument. Our cell-inhibition experiments showed that the initial softening is caused by MMP-mediated degradation of collagen, with relevance to breast cancer (Coussens et al. (2002); Luo et al. (2015); Mak et al. (2011); Valastyan and Weinberg (2011); Wolf et al. (2013)), whereas stiffening is associated with local alignment and densification of collagen fibers around the fibroblasts. The fibroblast activity for the initial density of 0.5 M cells/mL also involved an increase of loss tangent during incubation, though this did not have a spatial dependence. The ability to measure cell-size-scale loss tangent may be critical in breast-cancer research, since loss modulus is associated with progressed breast cancer of imaged patient tissue in vivo (Sinkus et al. (2007)) and breast-cancer cells in 3D cultures respond to alterations of the matrix’s loss-tangent-related mechanical plasticity (Wisdom et al. (2018)). This work paves the way for the use of systems that precisely quantify microscale viscoelasticity within 3D cell cultures for cancer-progression studies.

## Materials and methods

### 3D collagen matrices without and with fibroblasts

The main experiments using 1.0-mg/mL-concentration collagen, without and with fibroblasts, were based on a type 1 collagen with a batch concentration of $\simeq$3.0 mg/mL (Corning cat. num. 354236). The calibration of the micromanipulator 1 for these main experiments also used a concentration of 2.0 mg/mL, diluted from this batch concentration. The calibration of the micromanipulator 2 required a higher batch concentration of 8–11 mg/mL that was used for dilutions of 2.0, 4.0, and 6.0 mg/mL (Fisher Scientific cat. num. CB354249).

The gelation of collagen is temperature and pH dependent (Wild et al. (2013)). Thus, the collagen was kept on ice to retain a constant temperature while it was neutralized with 10X Dulbecco’s modified eagle media (DMEM) using a volume ratio of 1:9 between the collagen and 10X DMEM. For validation, the pH of the neutralized collagen was controlled using a pH meter. In order to prepare a desired concentration, the neutralized collagen was diluted using 1X DMEM (Corning cat. num. 354236).

For all micromanipulator experiments, 100 $\mu$L of the diluting 1X DMEM was replaced with two acqueous solutions: 50 $\mu$L of 10-$\mu$m-diameter magnetic microprobes (Sigma-Aldrich cat. num. 49664; diluted to 0.3 wt%), and 50 $\mu$L of 6-$\mu$m-diameter non-magnetic reference microprobes (Polysciences cat. num. 15714; diluted to 0.3 wt%). Similarly for experiments with cells, a volume containing the fibroblast cells replaced an equal volume of the diluting 1X DMEM (see further details in the following paragraphs). Finally, the collagen dilutions, without or with fibroblasts, were gelated within custom-made sample holders in an incubator for 40 min. The stability of the collagen mechanical properties was verified via macromechanical measurements using a rheometer.

The experiments with collagen only were immediately carried out after this incubation. The collagen matrices with fibroblasts and identical control matrices without fibroblasts, were supplied with fibroblast growth media and stored in an incubator for 24, 32, 48, and 72 h, prior the experiments.

### Fibroblast cell cultures

3T3 fibroblasts from mouse were cultured in a growth media consisting of 1X DMEM, and 10% and 1% volumetric dilutions of fetal bovine serum and Penicillin/Streptomycin, respectively. As the fibroblast cells reached 90% confluency, they were treated with 0.05% trypsin/EDTA, centrifuged for 5 min, and subsequently passaged onto cell-culture dishes at densities of 2–3 million cells/mL. The fibroblast growth media was changed every 2–4 days until embedding the cells into the collagen matrices. The main 3D-collagen-matrix experiments used an initial cell density of 0.5 million cells/mL. The complementary experiments used an increased initial cell density of 2.0 million cells/mL.

### Inhibition of fibroblasts’ MMP production

To inhibit the MMP production of the fibroblasts, the growth media of the fibroblast-containing collagen matrices was supplied with a 10 $\mu$M solution of GM6001 (Millipore cat. num. CC1010; 1.0 mg/mL, 2.5 mM, in DMSO), and subsequently incubated for 32 h. The protocol and the concentration were as in the similar inhibition studies (Sabeh et al. (2009); Schoumacher et al. (2010); Wisdom et al. (2018)).

### Macromechanical measurements using a (macro) rheometer

Macromechanical measurements of collagen matrices were made using a AR2000ex rheometer (TA instruments). Immediately after diluting with 1X DMEM, the collagen was deposited with a syringe directly onto the bottom plate of the rheometer, and a 25 mm flat parallel plate was brought down. Thus, a 25 mm disk of collagen was formed. Mineral oil (Sigma) was applied onto the edges of the disk to prevent drying. Macroscale viscoelastic properties at a frequency of f=0.05 Hz and a strain of 1 % were measured during the gelation of 40 min, until which |G| and $\phi$ reached an equilibrium.

### Micromechanical measurements using micromanipulators

Both micromanipulators consisted of two electromagnets with an outer diameter of 80 mm and length of 47 mm (Trafomic, Raisio, Finland). The micromanipulator 1 and 2 had an electromagnet inner diameter of 6.0 mm and 3.0 mm and corresponding 6.0-mm- and 3.0-mm-diameter cobalt–iron cores (Vacoflux 50, Vacuumschmelze, Hanau, Germany), respectively. The micromanipulator 1 and 2 were fixed on designated microscopes, Zeiss Axiovert 200M and Nikon Eclipse Ti2, respectively. The 3D collagen matrices were quantified in custom-made sample holders, fixed in the middle of the micromanipulator workspace (see Fig. 1d). The matrices were imaged using a 20X objective together with a Zeiss Axiocam 105 camera in Zeiss Axiovert 200M and an ORCA-Flash 4.0 LT+ camera in Nikon Eclipse Ti2.

Micromechanical measurements are carried out via the application of magnetic forces (Abbott et al. (2007)) onto the microprobes in the 3D collagen matrices. Each of the two electromagnets (see Figs. 1a–b) was driven by a custom-made amplifier using a bipolar 40 V power supply (Keysight cat. num. U8032A). A custom-made Labview software was used for the generation and control of sinusoidal currents, via a DAQ card (National Instruments cat. num. NI PCIe6341) connected to the amplifiers. The software was adapted based on the one used in Buttinoni et al. (2017). Based on scripts input into the software, accurate currents were fed to both electromagnets at a frequency of 1000 Hz. Specifically, the desired sinusoidal current sequences were defined. After carrying out a current sequence (ie. exerting forces F on the microprobes), the timing and current data, together with microscope images were recorded in the PC.

Controlled sinusoidal forces F were applied on the microprobes within 3D collagen matrices at a frequency of f=0.05 Hz (see Eq. 4). The displacement response of the magnetic microprobes to the forces was tracked, using Matlab-based image-processing algorithms with background subtraction and binarization, from the images recorded at 20 fps (see Eq. 5). In order to remove any mounting-related displacement noise during experiments, tracked displacements of the non-magnetic reference microprobes were subtracted from the displacement response.

### Statistical analysis

Each microscale viscoelasticity value ($|\text {G}|$ and $\phi$) is based on 2–5 repetitions using a probe, unless otherwise noted. A distinct statistical analysis was performed for absolute and relative viscoelasticity. For absolute viscoelasticity, mean viscoelasticity values of each sample were used for statistical analysis to account for inter-sample variation. For relative viscoelasticity, $|\text {G}|$ and $\phi$ were already normalized with the mean value of the sample or the control sample, thus, probe-based data was directly used for statistical analysis.

Viscoelasticity trends as a function of discrete incubation times were analyzed using linear regression and analysis of variance (ANOVA) (ie. ANOVA noted for Figs. 4a–d). The logarithmic transformation of the data enhanced the SD homogeneity, thus increased the statistical power of ANOVA. If no significant incubation time-dependent trend was found based on individual data points (ie. for loss tangent; see Figs. 4b), mean values of the data points for each incubation time were used. If no incubation time-dependent trend was found, the significance of the incubation time-independent increase was investigated. Lumped mean values of the experiments and their controls, independent of incubation time, were analyzed using unpaired one-sided t-test. Next, softening based on absolute-viscoelasticity values at 32 h incubation was statistically analyzed using unpaired one-sided t-test (see Figs. 4a–b). The corresponding relative-viscoelasticity values were analyzed using paired one-sided t-test (see Figs. 4c–d), which by definition involved pairing with the controls.

Relative-viscoelasticity trends for continously varying, cell–probe distances r, were analyzed using linear regression and Pearson’s correlation test (see Figs. 5a–b). For preprosessing, binning was used to minimize minor observation errors. Binning invervals of 15 $\mu$m were used, and the data was plotted to the mean value of each bin. Analyzing the logarithm of the data improved the statistical power of ANOVA (ie. via enhancing the SD homogeneity). If no significant trend was found (ie. for relative loss tangent; see Fig. 5b), the significance of the cell–probe distance-independent increase was investigated using unpaired one-sided t-test.

The tests for inhibiting fibroblasts’ MMP production via GM6001 treatment were first statistically analyzed using paired one-sided t-tests. Relative-viscoelasticity values of the matrix for the GM6001-treated and wild-type cells were compared. Then, a Bonferroni correction for multiple comparisons was applied to acquire the Pr values (see Fig. 6).

## Supplementary material

Supplementary material consists of supplementary figures on uniformity of magnetic-field gradients (see Fig. S1) and magnetic fields (see Fig. S2), accuracy of measurements (see Fig. S3), force calibration of the micromanipulators (see Fig. S4), rheometry-based viscoelasticity of collagen matrices (see Fig. S5), spatial variance of collagen viscoelasticity (see Fig. S6), and the effects of initial fibroblast density on microscale viscoelasticity (see Fig. S7).

## Supplementary Information

Below is the link to the electronic supplementary material.

Supplementary file1 (DOC 2.40MB)
