# Supplementary material for: Magnetic probe-based microrheology reveals local softening and stiffening of 3D collagen matrices by fibroblasts
Source: Biomed Microdevices. 2021 Apr 26;23(2):27. doi: 10.1007/s10544-021-00547-2 (PMC8076128; doi:10.1007/s10544-021-00547-2)
Supplement: Supplementary file 1 — Supplementary file1 (DOC 2.40MB) [file 10544_2021_547_MOESM1_ESM.pdf]

SUPPLEMENTARY MATERIAL

Magnetic-field gradient $\frac{\partial \mathbf{B}}{\partial x}$ uniformity in radial direction

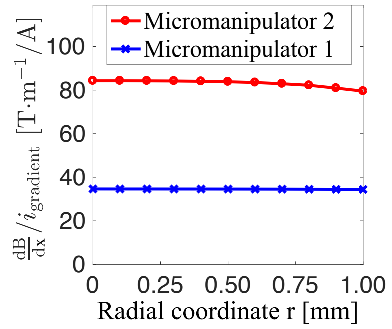

FIG. S1. Calibrated FEM simulation on magnetic-field gradient $\frac{\partial \mathbf{B}}{\partial x}$ dependence on radial position r, normalized by i_{gradient}. An axial coordinate of x=0 is used.

Magnetic field $\mathbf{B}_x$ uniformity in axial and radial directions

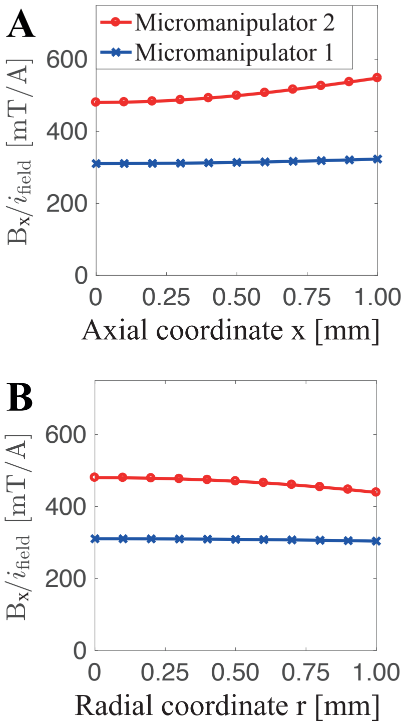

FIG. S2. Calibrated FEM simulation on magnetic field $\mathbf{B}_x$ dependence on **(A)** axial position x and **(B)** radial position r, both, normalized by i_{field}. **(A)** and **(B)** have a radial coordinate of r=0, and an axial coordinate of x=0, respectively.

Accuracy of measurements using microprobes

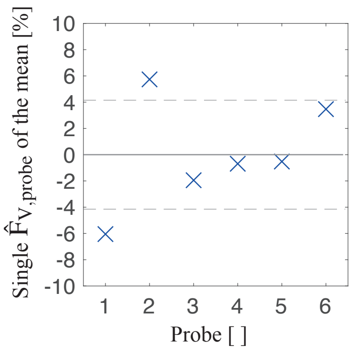

FIG. S3. Accuracy of single probe measurements. Solid line with 0% denotes for the mean value, while dashed line marks the SD.

Force calibration of the micromanipulators 1–2

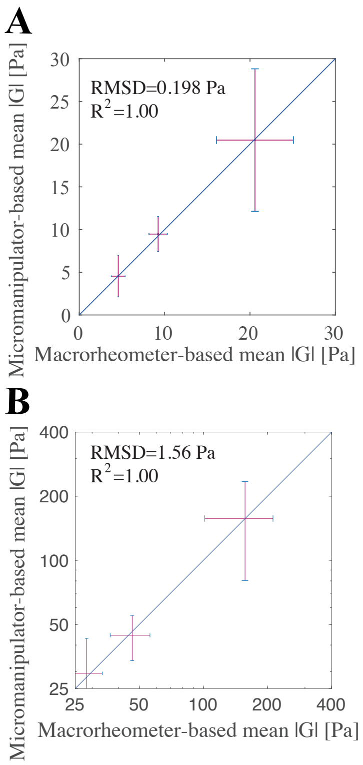

FIG. S4. Micromanipulator calibration against macrorheometry. **(A)** Micromanipulator 1 and **(B)** 2 compared against macrorheometer-based measurements, based on mean moduli |G|. Final volumetric force $\hat{\mathrm{F}}_{\mathrm{V,probe}}$ conversion is based on the slope of 1.00 for the relationship (plotted). The errorbars show the measurement SDs.

Viscoelasticity of collagen type 1 matrix

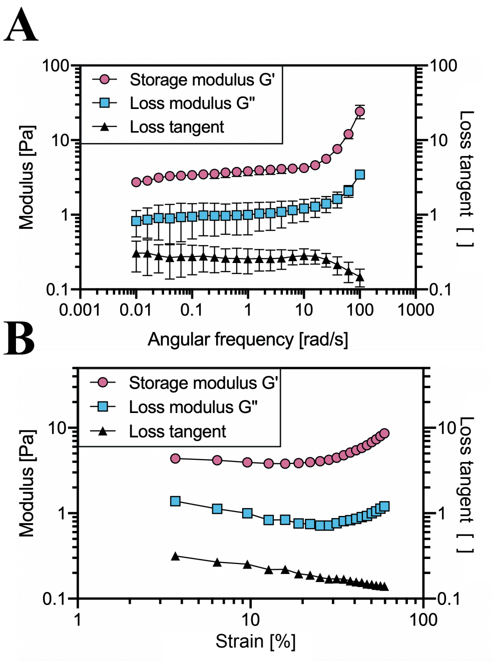

FIG. S5. Macrorheometer quantification of collagen type 1 at a concentration of c=1.0 mg/mL.
(A) Frequency dependence of viscoelasticity at a strain of 1 %. The means and SDs are based on 3 repetitions.
(B) Viscoelasticity versus strain at an angular frequency of 1 rad/s.

Spatial variance of the collagen matrix viscoelasticity

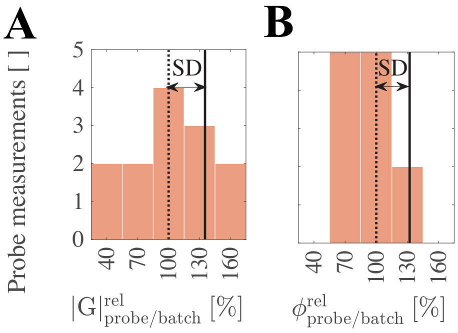

FIG. S6. Spatially varying viscoelastic properties at the microscale measured for collagen matrices at a concentration of c=1.0 mg/mL: **(A)** modulus-related $|G|^{rel}_{probe/batch}$ and **(B)** loss tangent-related $\phi^{rel}_{probe/batch}$. The SDs illustrate the spatially varying properties.

Effect of initial fibroblast density on collagen matrix viscoelasticity at the incubation of T=32 h

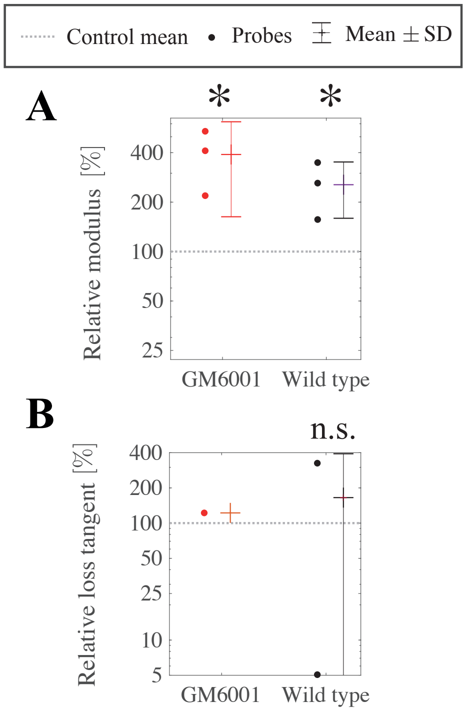

FIG. S7. Initial cell density affects on microscale viscoelasticity.
(A) Relative-modulus values of matrices with wild type and GM6001-treated cells are larger than the ones of the control at an increased initial fibroblast density of 2.0 M cells/mL (ie. both stiffer; *Pr < 0.05, paired t-test, n=3). **(B)** Matrices' loss tangent insignificantly varies from the one of control matrices (n.s. Pr > 0.20, n=2 for wild type).
